# Nurse Champions: Transforming Evidence

**DOI:** 10.15649/cuidarte.3745

**Published:** 2024-05-01

**Authors:** Lizeth N. Quiroga-Pico, Andrea M. Aceros-Lora, Tatiana M. Díaz-Castañeda, Lyda Z. Rojas

**Affiliations:** 1 Universidad Industrial de Santander, Bucaramanga, Colombia. E-mail: lizeth2191328@correo.uis.edu.co Universidad Industrial de Santander Universidad Industrial de Santander Bucaramanga Colombia lizeth2191328@correo.uis.edu.co; 2 Fundación Cardiovascular de Colombia, Floridablanca, Colombia. E-mail: andreaaceros@fcv.org Fundación Cardiovascular de Colombia Floridablanca Colombia andreaaceros@fcv.org; 3 Fundación Cardiovascular de Colombia, Floridablanca, Colombia. E-mail: tatianadiaz@fcv.org Fundación Cardiovascular de Colombia Floridablanca Colombia tatianadiaz@fcv.org; 4 Fundación Cardiovascular de Colombia, Universidad Industrial de Santander. E-mail: lyda.rojas@correo.uis.edu.co - lydarojas@fcv.org Universidad Industrial de Santander Universidad Industrial de Santander Colombia lyda.rojas@correo.uis.edu.co - lydarojas@fcv.org

Nursing practice in an organization result from the leadership processes developed for the profession. It is reflected in the performance of nurses in interprofessional teams and the quality of care they provide to patients^1^. Evidence-based practice (EBP) refers to decision-making in the development and delivery of health care services according to the best available research evidence, the experience of health care providers, and the values and preferences of patients. The adoption or implementation of EBP by organizations can lead to safer practices, better outcomes for individuals, and lower healthcare costs^2^. In the literature, "champions" have been identified as determinants and agents of change to ensure adoption within institutions^1^^, ^^3^.

The term champion first appeared in the literature in 1990, and its definition and characteristics have been built over time^1^, but what is a champion? A champion is a nurse who excels within an institution and is passionate about improving the quality of care^4^.

A good champion’s characteristics include good negotiation skills, advocacy, enthusiasm and energy to lead implementation, a thorough understanding of the initiatives and the local context, a positive approach, a solid ability to educate, and communication skills. Champions are characterized by their belief in, commitment to, and vocal and visible support of successful change initiatives; they are respected, credible, and valued by their peers^1^^, ^^3^.

In addition, champions are transformational leaders who can create, facilitate, and sustain change and broadly influence organizational culture, driving the adoption of best practices. In this sense, the roles of champions include advocating for initiatives within the work environment, motivating staff, participating in planning activities, and educating, training, and introducing staff to the initiatives, their importance, and their value. They also build relationships with key individuals, cross boundaries between services or units, spend one-on-one time with staff, recruit members for implementation, use data to persuade peers, and resolve issues that arise during EBP implementation^1^^, ^^5^. It has also been shown that champions tend to have longer tenure in the organization, more work experience, technical competence, and knowledge of the organization^6^.

The champions with the longest tenure are the ones with the most success. Because they are familiar with the risks and challenges associated with implementation, they can better build communication networks and understand the boundaries within which the organization operates and the need for change^6^. However, due to the multiple and diverse functions of the champion role, it is important to consider that these responsibilities may be associated with more than one nurse within the institution. The literature indicates that multiple champions must work simultaneously and in a coordinated manner in a single direction for implementation to be successful^1^.

As the term “champion” has evolved over time, and based on the different dynamics of an organization, the role of the champion has been adapted according to the service, unit, or area and the specific needs of these settings or the problem to be addressed^1^^, ^^5^. For example, some classifications of this role include: a) by its function within the organization (care champion, organizational change champion, administrative champion); b) by its related topic (hand washing champion, guidelines and protocol champion, innovation champion, EBP champion); and c) by a specific job position (nurse champion, educator champion, among others)^1^^, ^^5^.

It should be noted that a professional may self-identify as a champion or be chosen by his or her peers to assume this role, which may be informal but may also be formalized. Formal champions are typically appointed by a service coordinator to carry out a specific project for which they are trained. They are key because they are officially charged with finding and managing improvements. On the other hand, informal champions are nurses who develop, actively promote, and implement an innovative program or project within their unit without being formally appointed to do so; this role relies on their personal resources and influence but also implies greater flexibility and the opportunity to choose practical problems and solutions that do not have to follow standardized processes^4^^, ^^6^.

**Figure 1:**
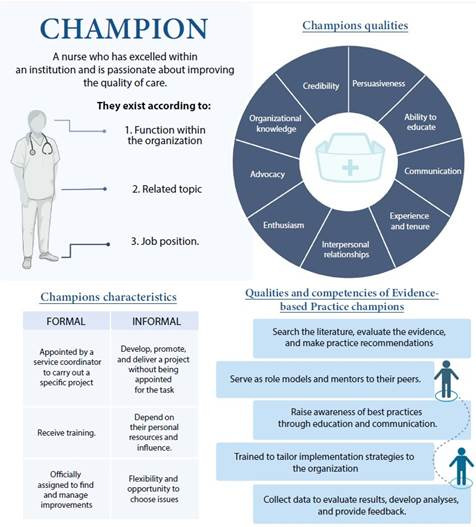
Definition, types, characteristics, and qualities of champions

The distinction between these types of champions is not intended to establish the superiority of one type over the other but to reflect the importance of making a conscious selection of these leaders based on their experience, tenure, knowledge, and capacity in the area of project development, ensuring the necessary resources and support, and motivating them to lead innovation projects^4^. Figure 1 summarizes the key characteristics of the champion role.

According to all of the preceding, EBP champions are nurses who promote the delivery of the best evidence-based care interventions, and the interpersonal relation inherent in their role is key to the successful implementation of EBP. Champions create awareness of best practices through educational strategies and act as mentors to nurses; they act as persuasive leaders for EBP implementation and are best able to tailor implementation strategies to the organization.

Champions are also facilitators of EBP sustainability in multiple contexts, and improved adherence to clinical best practices has been observed in studies only after champions are involved^4^^, ^^7^^, ^^8^. Some of the most important responsibilities of champions are education and communication. Champions often serve as role models and mentors to their peers, collect data to evaluate outcomes and develop analyses, provide feedback, and advocate for new ideas that benefit nurses and patients. Education is a critical component for EBP champions and promotes adoption. It is typically provided one-on-one with bedside nurses and interprofessional teams.

On the other hand, communication skills are needed to influence the adoption of EBP and to build relationships between professionals and services within the organization, which promotes a cultural change that sustains EBP^7^^, ^^8^.

It is important to consider that the role of the EBP champion requires a set of competencies and skills, such as searching the scientific literature, evaluating evidence, and making practice recommendations. These competencies are often not found in the professional profile of nurses or coordinators, which implies a barrier to the existence of EBP champions in organizations. Evidence suggests the need to engage nurses trained in EBP roles to become peer educators at all levels of care to strengthen nurses’ beliefs in EBP and provide them with the tools to transition to best practices successfully^8^^, ^^9^.

Finally, we would like to highlight two studies that used champions to perform EBP activities. The first is a study conducted in Canada that identified the influence of best practice champions in disseminating best practice guidelines through education with workshops, group education sessions, and interactive audio-visual learning resources, thereby advocating for the education of the entire workforce. These champions were also perceived as guideline experts and role models, working through a complex system of organizational groups and committees; they were the leaders of interdisciplinary teams to facilitate the dissemination of guidelines. They also audited, monitored, and documented changes to implement recommendations^10^.

The other study is about care champions who implemented a pain management protocol through stafftraining sessions and individualized education. These champions listened to feedback from their peers about ways to reduce the workload associated with protocol implementation. They also taught about pain-related diagnoses, typical and atypical pain responses, and pharmacologic management. They frequently met with service leaders to discuss the protocol and answer questions. They committed to maintaining compliance through personal and bulletin board reminders, incorporating a pain assessment tool into the system, and conducting audits. These nurses were responsible for organizing monthly interdisciplinary meetings to facilitate the use of the protocol and to address issues that arose^11^.

In conclusion, nurse champions are key transformational leaders and organizational change agents. Nurse champions have different characteristics and roles that vary depending on the needs and goals of the organization. Likewise, they may self-identify as champions or be selected by their peers; however, the institution must provide support and resources that encourage and foster their leadership in nursing practice. The purpose of this support is to ensure that the various challenges undertaken (assistive, administrative, educational, EBP, among others) successfully lead to sustainable outcomes over time and improve the quality of care.
